# Effects of Dietary Supplementation with Cocrystals of Thymol and Carvacrol on Quality, Nutrient Composition, and Oxidative Stability of Broiler Meat

**DOI:** 10.3390/foods13182899

**Published:** 2024-09-13

**Authors:** Yang Li, Changjin Li, Yunlong Zhang, Nadia Everaert, Luke Comer, Libo Huang, Ning Jiao, Xuejun Yuan, Weiren Yang, Shuzhen Jiang

**Affiliations:** 1 Key Laboratory of Efficient Utilization of Non-Grain Feed Resources (Co-Construction by Ministry and Province), Ministry of Agriculture and Rural Affairs, Shandong Provincial Key Laboratory of Animal Nutrition and Efficient Feeding, College of Animal Science and Technology, Shandong Agricultural University, Panhe Street 7, Tai’an 271017, China; li_yang@sdau.edu.cn (Y.L.); 17664393914@163.com (C.L.); 18853843376@163.com (Y.Z.); huanglibo@sdau.cn (L.H.); jiaoning@sdau.cn (N.J.); wryang@sdau.cn (W.Y.); 2 Division of Animal and Human Health Engineering, Department of Biosystems, KU Leuven, Kasteelpark Arenberg 30, 3001 Heverlee, Belgium; nadia.everaert@kuleuven.be (N.E.); luke.comer@kuleuven.be (L.C.); 3 College of Life Sciences, Shandong Agricultural University, Daizong Street 61, Tai’an 271018, China; xjyuan@sdau.cn

**Keywords:** antioxidant, cocrystal, essential oil, meat quality, nutritional values

## Abstract

Consumer demand for high-quality meat has increased. This study aimed to investigate the potential application of cocrystals of thymol and carvacrol in broilers for high-quality meat production. Eight hundred 1-day-old chicks were assigned to four groups fed diets supplemented with 0, 40, 60, and 80 mg/kg of Crystal EO^®^ (CEO), containing 25% cocrystals of thymol and carvacrol in a 42-d feeding trial. The results showed that dietary CEO supplementation decreased the muscle fiber diameter and increased the muscle fiber density, glycogen content, L*_45 min_ value, and proportion of α-linolenic acid in the breast muscle; dietary 40 and 60 mg/kg of CEO decreased the lactate content, MDA concentration, cooking loss, shear force, and thrombogenicity index and increased the proportion of lauric acid in the breast muscle; dietary 60 and 80 mg/kg of CEO increased the glucose content, total superoxide dismutase, and total antioxidant capacity levels of breast muscle. Citrate synthase activity, free radical scavenging capacity, pH_24 h_ and a*_45 min_ values, and the cystine content in the breast muscle were especially higher in the 60 mg/kg CEO group compared to the control group. Collectively, dietary CEO supplementation improved meat quality and nutritional values and enhanced the antioxidant capacity of broiler meat, with 60 mg/kg of CEO having the greatest effect.

## 1. Introduction

Nowadays, chicken is one of the preferred meats among consumers worldwide, given its affordable price and high-protein and low-fat content [1]. Over the past 60 years, driven by the need to improve broiler production efficiency to fulfill market demands, the genetic selection of broilers has concentrated predominantly on production traits, namely, growth rate and feed conversion, leading to a decrease in the meat quality of broilers [2]. Nevertheless, market demands have shifted over recent years, with high-quality meat and meat products gaining increased consumer interest, alongside the increased demand for convenient, minimally processed, and ready-to-eat meat products. This relatively novel notion of all-natural and clean-label products combined with economic favorability and convenience poses a challenge to producers and manufacturers alike in generating high-quality chicken meat to satisfy these demands.

Essential oils (EOs) are highly concentrated mixtures consisting of volatile, lipophilic, and predominantly terpenoid compounds. As secondary metabolites produced by plants, EOs can, in turn, be extracted from a range of vegetation, including flowers, fruits, seeds, leaves, and roots [3]. Chicken, in comparison to some other meats, is particularly susceptible to oxidative deterioration given its high concentration of polyunsaturated fatty acids (PUFAs), causing a deterioration in meat quality [1]. Numerous studies on broiler chicken production have indicated that dietary EO supplementation can alter the meat’s fatty acid composition and enhance the antioxidative status, in turn improving meat quality [3]. Thymol and carvacrol are the two major components of oregano oil and are known to have many biological effects, including antioxidant, anti-inflammatory, antibacterial, and antiviral properties [4]. Therefore, they are considered a safe and beneficial alternative to chemicals in many applications, including animal production, alternative medicine, and food preservation [5]. Furthermore, the combination of thymol and carvacrol has shown the best antioxidant activity due to their synergistic interactions compared with the individual addition of either thymol or carvacrol [6]. Luna et al. [7] have demonstrated that the supplementation of 150 mg/kg of thymol or carvacrol in broiler diets delays lipid oxidation in meat, and both can be regarded as beneficial additives for application in the poultry industry to improve meat quality. However, EOs, including thymol and carvacrol, are typically characterized as having very low or no solubility in water, a low melting point, and high volatility, and they tend to undergo changes such as thickening, darkening, and exhibiting acidic reactions when exposed to air for an extended period of time [8]. As such, the difficulty of low oral bioavailability arises and presents a challenge to the maintenance of biological activity.

Cocrystal technology is an emerging and effective method used in the pharmaceutical industry to improve drug solubility, stability, and bioavailability [9]. Cocrystals are multicomponent, crystalline structures that can be made from a range of chemicals in a stoichiometric ratio. Previous research has demonstrated that interactions between an EO molecule and suitable partner molecules could establish a strong intermolecular network, thus stabilizing the liquid ingredient of EOs in a solid form [5]. Cocrystallization possesses significant potential for enhancing the physicochemical characteristics of active constituents while preserving their molecular structure and bioactivity [9]. However, to our knowledge, the scientific literature evaluating the application of cocrystals of thymol and carvacrol in broilers is currently scarce. Therefore, it is meaningful to evaluate the effects of cocrystals of thymol and carvacrol on meat quality, nutrient composition, and oxidative stability in broiler meat.

Based on the above, our study aimed to explore the effects of dietary supplementation with different levels of cocrystals of thymol and carvacrol on meat quality, amino acid, and fatty acid profiles, as well as the oxidative stability of broiler breast muscle, providing support for the application of cocrystals of thymol and carvacrol to improve meat quality in poultry production.

## 2. Materials and Methods

### 2.1. Cocrystals of Thymol and Carvacrol

The cocrystals of thymol and carvacrol were provided by Cocrystsal Health Co., Ltd. (Jiashan, China), commercially named Crystal EO^®^ (CEO). CEO is a mixture in equal parts of the cocrystal of thymol with L-proline (molar ratio 1:1) and the cocrystal of carvacrol with L-proline (molar ratio 1:1). Thymol and carvacrol are extracted from oregano oil, and the effective content is 25% in the CEO. Calcium phosphate is used as a premix carrier in the CEO.

### 2.2. Experimental Design and Management

A total of eight hundred 1-day-old Arbor Acres chicks, comprising an equal number of males and females, were assigned to four treatment groups randomly, with eight replicates per group and twenty-five broilers per replicate (cage), in a 42-d trial. The broilers were fed a basal diet supplemented with either 0 (control), 40, 60, or 80 mg/kg of CEO, denoted as CEO0 (control), CEO40, CEO60, and CEO80, respectively. The formulation of the basal diets (Table 1) followed the nutritional guidelines recommended by the National Research Council for broiler chickens, in alignment with a 3-phase feeding program (1–14 d, 15–28 d, and 29–42 d). During the trial, the broilers were housed in a three-level metal chicken coop located in a temperature-controlled room with constant lighting, and they had free access to feed and water throughout.

### 2.3. Sample Collection

After 12 h of fasting, a total of 32 broilers (8 birds/treatment; one broiler in each replicate with a body weight close to the average of the replicate) were chosen for sample collection at the end of the experiment. The selected broilers were euthanized via cervical dislocation. Within 10 min postmortem, a tissue specimen (0.5 × 0.5 × 2 cm^3^) of the left breast muscle of the broilers was cut and fixed in 4% paraformaldehyde solution at room temperature; the remaining part of the left breast muscle was promptly collected and stored in air at 4 °C to assess changes in breast muscle quality of broilers. Additionally, the entire right breast muscle of the broilers was also collected, one part being stored at −20 °C to determine muscular chemical composition, antioxidant capacity parameters, and glucose metabolism and other part being stored at −80 °C for the analysis of amino acid and fatty acid profiles.

### 2.4. Meat Quality

At 45 min and 24 h postmortem, the pH values of each sample were measured using a pH Star 6.05 machine (Matthäus, Bavaria, Germany). Concurrently, the color parameters, including L* (lightness), a* (redness), and b* (yellowness) values, were determined using a Colorimeter (CR410, Minolta, Tokyo), as described in previous studies [10,11]. Water-holding capacity was evaluated through drip loss [12], cooking loss [12], and thawing loss [13]. Shear force measurements were conducted on cooked breast muscle samples, which were cut parallel to the longitudinal orientation of the myofibers after being cooled to 4 °C. The shear force of each sample was measured three times using a TA-XTPlus Texture Analyzer (Stable Micro Systems, Godalming, UK) fitted with a 30 kg load cell, and the greatest force value was recorded as the shear force [14,15].

### 2.5. Muscle Morphological Analysis

The left breast muscle tissues were dehydrated through a graded series of ethyl alcohol solutions with increasing concentrations and subsequently embedded in liquid paraffin following a 24 h fixation in 4% paraformaldehyde, as previously described [10]. Tissue sections, 5 µm in thickness, were then stained with hematoxylin and eosin (H&E) and examined using an Olympus BX-51 digital microscope equipped with an Olympus DP73 digital camera (Olympus, Tokyo, Japan) and CellSens Imaging software (Olympus America, Melville, NY, USA, https://lifescience.evidentscientific.com.cn/en/software/cellsens/, accessed on 4 September 2024). The diameters of 30 completely visible muscle fibers were measured, and muscle fiber density was assessed in five randomly selected regions from each sample using 400× photomicrographs. These measurements were calibrated with Image-Pro Plus 4.5 software (MediaCybernectics, Bethesda, MD, USA) [10]. Muscle fiber density was operationally defined as the number of muscle fibers per unit cross-sectional area.

### 2.6. Chemical Composition of the Breast Muscle

To quantify the moisture, crude fat, and crude protein content in the meat, approximately 40 g of the right breast muscle sample was weighed, sliced, and placed on a ceramic plate for reweighing. Subsequently, the ceramic plate was placed in a laboratory lyophilizer (Free Zone 6, Labconco Corp., Kansas City, MO, USA) at −50 °C for 48 h. The moisture content was determined by calculating the weight difference between the initial and dried muscle samples (934.01; AOAC, 2006). The dried muscle sample was then ground into a powder for subsequent analysis to determine the crude protein and crude fat content according to AOAC (2006) Official Method 990.03 and 920.39 A, respectively.

### 2.7. Glucose Metabolism in Breast Muscle

The content of glycogen, glucose-6-P, glucose, and lactate was quantified using commercially available kits from Nanjing Jiancheng Bioengineering Institute (Nanjing, China), adhering strictly to the manufacturer’s protocols. Citrate synthase activity was measured using an ELISA kit from Jiangsu Meimian Industrial Co., Ltd. (Yancheng, China), following the procedure outlined below. Briefly, muscle samples were homogenized in ice-cold saline solution (1:9, *w*/*v*) and subsequently centrifugated at 12,000× *g* for 10 min at 4 °C to obtain supernatants. The supernatants (50 μL) and diluted standard solutions were added to the corresponding microplates. Following a 30 min incubation at 37 °C, the microplates were washed five times, and 50 μL of HRP-Conjugated Reagent was added to each well. After another 30 min incubation at 37 °C and an additional five washes, chromogenic agents were applied for 10 min at 37 °C, followed by the addition of Stop Buffer. The absorbance of each well was measured within 15 min, and citrate synthase activity was quantified using a standard curve derived from the standard solutions.

### 2.8. Antioxidant Capacity Parameters in Breast Muscle

Breast muscle tissue samples were homogenized in ice-cold 0.9% sodium chloride (1:10, *w*/*v*) and subsequently centrifugated at 4000 rpm for 10 min to obtain the supernatant. The levels of malondialdehyde (MDA), glutathione peroxidase (GSH-Px), total superoxide dismutase (T-SOD), and total antioxidative capacity (T-AOC) in the supernatant were determined using commercial kits purchased from Nanjing Jiancheng Bioengineering Institute [16]. The levels of T-AOC, T-SOD, GSH-Px, and MDA in the breast muscle were normalized to the total protein content of each sample. The free radical-scavenging capacity of the breast muscle was assessed using the 2,2′-azobis-(3-ethylbenzothiazoline-6-sulfonic acid) (ABTS) and 2,2-diphenyl-1-picrylhydrazyl (DPPH) methods [11] with kits obtained from Jiangsu Aidisheng Biological Technology Co., Ltd. (Yancheng, China).

### 2.9. Amino Acid Profiles in Breast Muscle

The amino acid profile of breast muscle samples was detected using Waters ACQUITY I-CLASS UPLC System (Waters Corporation, Milford, MA, USA) with Waters Xevo TQ-S tandem quadrupole mass spectrometer (UPLC-MS/MS) at Beijing Hexin Technology Co., Ltd. (Beijing, China). Following the methodology outlined in a previous study [11], 30 g samples of breast muscle were weighed and digested with 2 mL of 6 mol/L HCl at 110 °C for 22 h. Subsequently, 50 µL of the resulting filtrate was extracted and subjected to evaporation until complete dryness. The dried sample was then re-dissolved in 1000 µL of double-distilled water (ddH_2_O). From this re-dissolved solution, 20 µL was transferred into a 1.5 mL centrifuge tube, to which 5 µL of an internal standard for each amino acid (Sigma-Aldrich, St. Louis, MO, USA) and 40 µL of isopropanol (0.1% formic acid) were added. The solution was subjected to vortex mixing for 5 min and subsequently centrifuged at 12,000 rpm for 10 min at 4 °C. An aliquot of 10 µL of the supernatant was transferred into a 1.5 mL centrifuge tube, followed by the addition of 70 μL borate buffer and 20 μL of AccQ·Tag derivative reagent (Kairos amino acid kit). The resulting mixture was then shaken for 10 s. Following an incubation for 1 min at 25 °C, the centrifuge tube was heated for 10 min at 55 °C. Finally, the mixture was diluted with 400 μL of ddH_2_O and transferred into autosampler vials for subsequent amino acid determination. The chromatographic separation was achieved using an ACQUITY UPLC HSS T3 column (2.1 × 150 mm^2^, 1.8 µm particle size) with a water (containing 0.1% formic acid)–acetonitrile linear gradient at a flow rate of 0.5 mL/min in a 4.5 min turn-around time.

### 2.10. Fatty Acid Profiles in Breast Muscle

The fatty acid content of breast muscle samples was analyzed using a GC–MS platform provided by Beijing Hexin Technology Co., Ltd. In brief, 50 mg of thawed muscle samples was weighed and homogenized. Subsequently, 3 mL of hexyl hydride and 3 mL of potassium hydroxide–methanol solution (0.4 mol/L) were sequentially added to the homogenate, with each addition followed by shaking at 50 °C for 30 min. The samples were then allowed to equilibrate to room temperature before being mixed with 1 mL of ddH_2_O. Finally, 90 μL of the supernatant was mixed with 10 μL of internal standard (100 μg/mL methyl nonadecanoate) and subjected to chromatography analysis using an Agilent 8860 gas chromatography system coupled to an Agilent 5977C mass selective detector (Agilent Technologies, Santa Clara, CA, USA). A G3903-63011 model DB-FastFAME (Agilent Technologies) was employed for the separation of fatty acid components. Quantification of individual fatty acids was achieved by measuring peak areas, with results expressed as a percentage of the total fatty acids. The mean content of each fatty acid was utilized to ascertain the total saturated fatty acids (SFA) and the unsaturated fatty acids (USFA), which encompassed both monounsaturated fatty acids (MUFA) and PUFA. Based on the results, the USFA:SFA ratio, ratio between hypocholesterolemic and hypercholesterolemic fatty acids (h/H), atherogenocity index (AI), and thrombogenicity index (TI) in the breast muscle were calculated according to the equations described by Anacleto et al. [17].

### 2.11. Statistical Analysis

Each individual broiler was designed as the experimental unit for the analysis of variables. The data were subjected to statistical analysis using the PROC GLM procedure of SAS (Institute Inc., Cary, NC, USA) with a 5% probability of error by using Duncan’s multiple comparison test. The normality of the data was assessed using the Shapiro–Wilk statistical test (W > 0.05). Results are expressed in the figures as the mean ± standard error and in the tables as means and standard error of the mean. Statistically significant differences were regarded at *p* < 0.05.

## 3. Results

### 3.1. Effects of CEO Supplementation on Antioxidant Capacity of Breast Muscle

**Figure 1:**
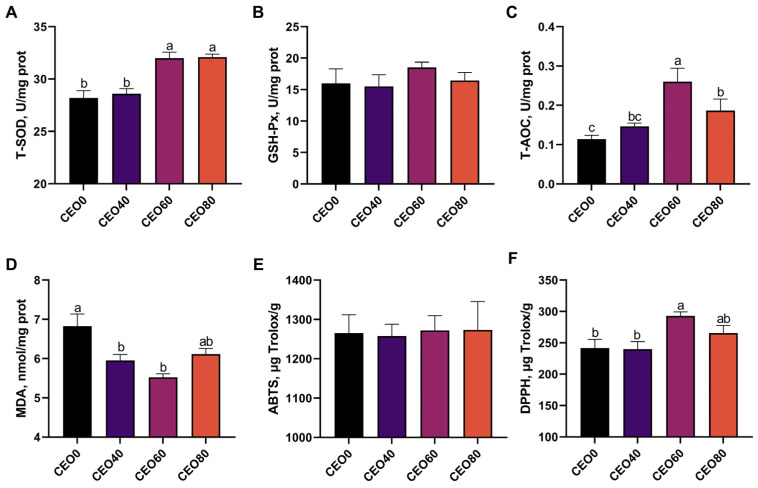
Effects of dietary supplementation with cocrystals of essential oils (CEO) on antioxidant capacity of the breast muscle in broilers. (**A**) T-SOD; (**B**) GSH-Px; (**C**) T-AOC; (**D**) MDA; (**E**) ABTS; (**F**) DPPH. CEO0, CEO40, CEO60, and CEO80 were the basal diet supplemented with 0, 40, 60, and 80 mg/kg cocrystals of thymol and carvacrol, respectively. Values are expressed as the mean and standard error of the mean (SEM). *n* = 8. ^a,b,c^ Means with different lowercase letters differ significantly among the four treatments (*p* < 0.05).

As shown in Figure 1, broilers in the CEO60 and CEO80 groups had significantly higher T-SOD activity in the breast muscle than broilers in the CEO0 group and CEO40 groups (*p* < 0.05). The broilers in the CEO60 group exhibited the lowest MDA concentration and the highest T-AOC and DPPH levels in the breast muscle among the four treatment groups. Moreover, the breast muscle T-AOC level in the CEO60 group was significantly higher than those of the other three groups (*p* < 0.05), and that of the CEO60 group was significantly higher than in the CEO0 group (*p* < 0.05). A significantly lower MDA concentration in breast muscle was found in the CEO40 and CEO60 groups relative to the CEO0 group (*p* < 0.05). The breast muscle DPPH level in the CEO60 group was significantly higher than that in the CEO0 and CEO60 groups (*p* < 0.05).

### 3.2. Effects of CEO Supplementation on Breast Muscle Meat Quality

As shown in Table 2, compared with the control group, dietary CEO supplementation significantly increased the L*_45 min_ value of breast muscle (*p* < 0.05); 60 mg/kg of CEO supplementation significantly increased the pH_24 h_ value and a*_45 min_ value of breast muscle (*p* = 0.033); 40 mg/kg and 60 mg/kg of CEO supplementation significantly decreased the cooking loss and shear force of breast muscle (*p* < 0.05). The CEO40 group showed a significantly lower a*_45 min_ value than the CEO60 group (*p* < 0.05) and significantly lower cooking loss than the CEO80 group (*p* < 0.05).

### 3.3. Effects of CEO Supplementation on Breast Muscle Fiber Characteristics

**Figure 2:**
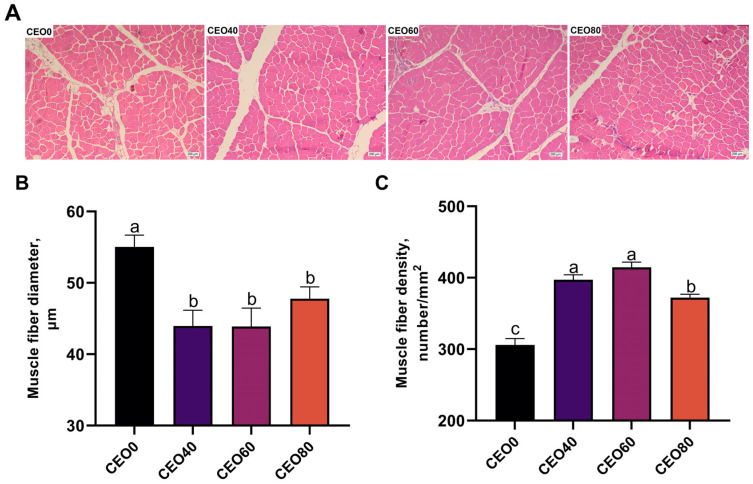
Effects of dietary supplementation with cocrystals of essential oils (CEO) on breast muscle fiber characteristics in broilers. (**A**) Hematoxylin and eosin photomicrographs of breast muscle (40× magnification); (**B**) Muscle fiber diameter; (**C**) Muscle fiber density. CEO0, CEO40, CEO60, and CEO80 indicate the basal diet supplemented with 0, 40, 60, and 80 mg/kg cocrystals of thymol and carvacrol, respectively. Values are expressed as the mean and standard error of the mean (SEM). *n* = 8. ^a,b,c^ Means with different lowercase letters differ significantly among the four treatments (*p* < 0.05).

As shown in Figure 2, compared with the control group, CEO supplementation significantly decreased the breast muscle fiber diameter (*p* < 0.05) and significantly increased the breast muscle fiber density (*p* < 0.05). In addition, broilers in the CEO40 and CEO60 groups had significantly higher breast muscle fiber densities than broilers in the CEO80 group (*p* < 0.05).

### 3.4. Effects of CEO Supplementation on Glucose Metabolism in Breast Muscle

**Figure 3:**
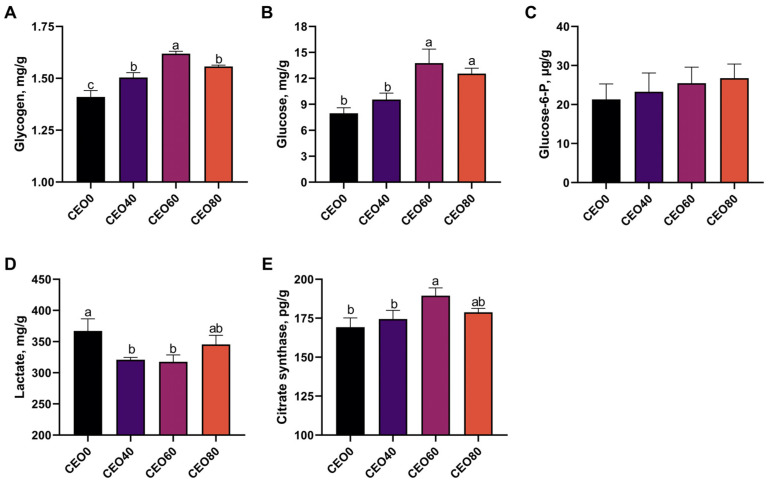
Effects of dietary supplementation with cocrystals of essential oils (CEO) on glucose metabolism in the breast muscle in broilers. (**A**) Glycogen; (**B**) Citrate synthase; (**C**) Glucose-6-P; (**D**) Lactate; (**E**) Citrate synthase. CEO0, CEO40, CEO60, and CEO80 indicate the basal diet supplemented with 0, 40, 60, and 80 mg/kg cocrystals of thymol and carvacrol, respectively. Values are expressed as the mean and standard error of the mean (SEM). *n* = 8. ^a,b,c^ Means with different lowercase letters differ significantly among the four treatments (*p* < 0.05).

As shown in Figure 3, the glycogen content in the breast muscle was significantly increased by CEO treatment (*p* < 0.05), with the CEO60 group shown to have a significantly higher glycogen concentration than the CEO40 and CEO80 groups (*p* < 0.05). Compared with the CEO0 group, the glucose content of breast meat in the CEO40 and CEO60 groups was significantly increased (*p* < 0.05), while the lactate content was significantly decreased (*p* < 0.05). Moreover, citrate synthase activity in the CEO60 group was significantly higher than that of the CEO0 and CEO40 groups (*p* < 0.05).

### 3.5. Effects of CEO Supplementation on the Chemical Composition of Breast Muscle

As shown in Table 3, the crude protein content in the breast muscle in the CEO80 group was the highest, and significantly higher than that in the CEO0 group (*p* = 0.021). There were no significant differences in the moisture and crude fat content in breast muscle among the four treatments (*p* > 0.05).

### 3.6. Effects of CEO Supplementation on the Amino Acid Profile of Breast Muscle

The amino acid profile of breast muscle is shown in Table 4. The CEO60 group exhibited a significantly higher cystine content in the breast muscle compared to both the CEO0 and CEO80 groups (*p* < 0.01). No significant differences were observed in other amino acid content in breast muscle among the four treatments (*p* > 0.05).

### 3.7. Effects of CEO Supplementation on the Fatty Acid Profile of Breast Muscle

The fatty acid profile of breast muscle is displayed in Table 5. The CEO40 and CEO60 groups exhibited a significantly higher proportion of lauric acid (C12:0) in breast muscle relative to the CEO0 group (*p* < 0.05), and broilers in the CEO40 group showed a significantly higher C12:0 proportion in the breast muscle than broilers in the CEO80 group (*p* < 0.05). Dietary CEO supplementation significantly increased the proportion of α-linolenic acid (C18:3n3) in the breast muscle compared with the control group (*p* = 0.030). Moreover, the CEO40 and CEO60 groups showed significantly higher total USFA and significantly lower TI values compared with the CON and CEO80 groups (*p* < 0.05). 

## 4. Discussion

In the present study, dietary supplementation with 60 mg/kg of CEO increased the breast meat a* value at 45 min postmortem. Hernández-Coronado et al. [14] also demonstrated that dietary supplementation with Mexican oregano EO in drinking water increased the a* value of breast meat in chickens. The color of meat serves as a visual indicator of its freshness and quality, significantly influencing consumer purchasing decisions. Research has indicated that the a* value of meat is correlated with alterations in the chemical properties of myoglobin [12]. Myoglobin is particularly susceptible to oxidation, which results in the transformation of red oxymyoglobin to metmyoglobin, thereby reducing the a* value of the meat [18]. Phenolic compounds can donate H-atoms from their hydroxyl groups to react with peroxyl radicals, forming stabilized phenoxyl radicals and thereby terminating chain reactions of lipid peroxidation. Both thymol and carvacrol were reported to possess DPPH radical-inhibitory activity [19]. Luna et al. [7] showed that adding 150 mg/kg thymol or carvacrol to a broiler diet decreased the MDA concentration and suppressed lipid oxidation in meat, which might be related to the increased antioxidant enzyme activity by the thymol and carvacrol supplementation [20]. Nevertheless, Llana-Ruiz-Cabello et al. [6] demonstrated that at higher concentrations of thymol, carvacrol, and its mixture resulted in oxidative stress, highlighting the need to optimize the concentration of thymol, carvacrol, and its mixture for applications as antioxidants. In our study, 60 mg/kg of CEO supplementation also decreased the MDA concentration in broiler meat. MDA, recognized as a marker of oxidative stress and antioxidant status, is one of the terminal products of PUFA peroxidation within cells [11]. The observed reduction in the MDA concentration in the CEO60 group suggested diminished lipid peroxidation in the meat, which can be attributed to elevated levels of T-SOD and T-AOC levels, as well as enhanced free radical-scavenging capacity (represented as an increased DPPH level). These findings also implied that CEO may extend the duration of red coloration and delay the formation of metmyoglobin, thereby postponing meat color deterioration. Previous research on lambs demonstrated that dietary supplementation with oregano EO (62.7% carvacrol concentration) increased the a* value and prolonged the storage period of *longissimus lumborum* muscle [21]. In our study, we observed that CEO supplementation elevated the L* value at 45 min postmortem. Similarly, Kiyma et al. (2017) reported that supplementing broiler diets with 24 and 48 mg/kg of lavender EO increased the L* value of breast meat. Generally, meat color appears lighter when more water exudes onto the meat surface, with higher L* values typically associated with increased water content in the meat [22]. However, we found that dietary supplementation with 40 and 60 mg/kg of CEO decreased cooking loss of the breast meat in this study. Consistently, Galli et al. [23] conducted a study to investigate the impacts of phytogenic compounds, specifically carvacrol and thymol (100 mg/kg), on the quality of broiler breast meat. These findings showed a significant enhancement in the water-holding capacity of the meat compared to the control group. A prior investigation revealed that dietary supplementation with 100 mg/kg of oregano extract (containing10% carvacrol and 31% thymol) resulted in a 64.8% increase in carvacrol and a 55.2% increase in thymol content in broiler breast meat over a 42-day period [24]. Consequently, it is hypothesized that the observed increase in the L* value of breast meat at 45 min postmortem, induced by CEO supplementation, may be attributed to the adhesion and permeation of EO into the breast muscle. Experimental evidence showed that increasing the antioxidant capacity of meat could enhance its water-holding capacity by increasing proteolysis [3]. Above all, 60 mg/kg of CEO supplementation improved the redness and water-holding capacity of broiler meat by enhancing its antioxidative capacity.

In this study, we found that adding 60 mg/kg of CEO to the broiler diet elevated the pH value of breast meat at 24 h postmortem. The pH value is a critical parameter for evaluating meat superiority and inferiority, as it partially indicates the rate of glycolysis [3]. A rapid decrease in pH is typically associated with an increased rate of glycolysis and subsequent lactate accumulation [11]. In this study, the CEO60 group exhibited higher levels of glycogen and glucose, along with a lower lactate content in breast meat, compared with the CEO0 group. Similarly, Jahani et al. [25] found that carvacrol and thymol could affect cellular energy metabolism by inhibiting glycolysis. We also observed that a dietary addition of 60 mg/kg of CEO increased citrate synthase activity in the breast muscle in the present study. Citrate synthase, a pivotal mitochondrial enzyme, catalyzes the synthesis of citrate from acetyl-CoA and oxaloacetate within the mitochondrial matrix, playing a crucial role in energy generation via the tricarboxylic acid cycle [26]. Its enzymatic activity is a validated biomarker for evaluating mitochondrial density and oxidative capacity in muscle tissue [27]. It was reported that the preservation of mitochondrial integrity and functionality, particularly within the initial hours post-slaughter, can significantly influence the progression of postmortem metabolism, thereby affecting the muscle-to-meat-transition process and meat quality [28,29]. Mitochondrial activity directly affects the development and stability of meat color and inhibits postmortem pH reduction by regulating sarcoplasmic calcium homeostasis via influx and efflux systems within the mitochondrial membrane [28]. Moreover, citrate synthase is released from meat tissue following cell membrane damage caused by ice crystal formation, and reduced citrate synthase activity in muscle is typically associated with the microbial spoilage of meat [30]. Zhang et al. [29] found that dietary resveratrol supplementation increased the pH_24 h_ value and citrate synthase activity of breast meat. Therefore, the elevated pH value of breast meat at 24 h postmortem via 60 mg/kg of CEO supplementation was attributed to the decreased glycolysis rate and increased citrate synthase activity in this study.

In our study, supplementation with 40 and 60 mg/kg of CEO in the diet decreased the shear force of broiler breast meat, which was in agreement with the results of Rimini et al. [31] and Angelovicova et al. [32]. Meat tenderness is recognized as the paramount quality parameter affecting consumers’ ultimate satisfaction and is typically assessed objectively through shear force measurements. The characteristics of muscle fiber, such as its diameter and density, significantly impact the meat shear force [33]. In the current study, broilers fed diets supplemented with CEO40 and CEO60 exhibited a reduction in muscle fiber diameter and an increase in muscle fiber density compared to those fed a diet without CEO supplementation. Previous research demonstrated that thinner muscle fibers and increased fiber density usually accompanied better meat tenderness [33]. Therefore, our results suggested that CEO supplementation caused an increase in meat tenderness and, consequently, an improvement in palatability. However, the mechanisms underlying the improvement in muscle fiber characteristics through CEO supplementation remain unclear and require further investigation.

The amino acid and fatty acid composition of meat significantly influences its nutritional value and flavor, while also playing a vital role in human health and physiological function [29,34]. In the present study, 80 mg/kg of CEO supplementation increased the crude protein content of breast meat. Previous studies have similarly shown that the addition of thymol or carvacrol in the diet can enhance protein metabolism and promote protein deposition, thereby increasing the crude protein content in the breast meat of broilers [35,36]. In particular, 60 mg/kg of CEO dietary supplementation was shown to increase the cystine content in breast meat. Cystine, a sulfur-containing amino acid, plays a crucial role as a precursor in the development of meat flavor. It is known to contribute a meat-like, sweet, and sulfurous taste by reacting with sugars, thereby influencing the overall flavor profile of meat [37]. Moreover, cystine, serving as the primary substrate for the synthesis of the antioxidant glutathione, has various benefits, such as antioxidation, enhancement of cellular redox effects, and regulation of mitochondrial function and structure [38]. Consequently, the increased cystine content may be a contributing factor to the improved texture observed in the breast meat of the CEO60 group in this study. In addition, supplementation with 40 and 60 mg/kg of CEO resulted in increased proportions of C12:0, C18:3n3, and total USFA in the breast meat, suggesting that the CEO might influence the biosynthesis of fatty acids in broilers. Starčević et al. [34] reported that supplementing the broiler diet with thymol led to an increase in C18:3n3 content in the meat. Similarly, Galli et al. [23] showed that supplementation with a combination of herbal components (curcumin, carvacrol, thymol, and cinnamaldehyde) elevated C18:3n3 levels and decreased total SFA levels in chicken breast meat. The medium-chain fatty acid C12:0, known as lauric acid, exhibits potent antioxidant, antibacterial, and antidiabetic properties and has been utilized as a food additive and supplement in various formulations [39]. Hoa et al. [40] demonstrated that the application of a chitosan coating containing C12:0 significantly extended the shelf-life of aerobically packaged beef steaks for over 21 days during refrigerated storage. Consequently, the increased proportion of C12:0 in the meat might also contribute to the improvement in the physical characteristics of breast meat in this study. It is well known that carvacrol and thymol, serving as substances with strong antioxidative properties, probably block the oxidation of lipids, which may be the reason for the increased C12:0 and USFA content in the breast muscle via CEO supplementation in this study. Furthermore, an expanding body of evidence underscores the association between the proportional composition of fatty acids in meat and human health [3]. Specifically, the fatty acid C18:3n3, known as α-linolenic acid, is an essential member of the omega-3 series that cannot be synthesized endogenously by the human body and must be obtained through the regular dietary intake of foods containing C18:3n3 [41]. This fatty acid has demonstrated a range of beneficial effects, including cardiovascular protection, cholesterol reduction, anti-cancer properties, anti-inflammatory actions, anti-osteoporotic benefits, neuroprotection, and antioxidative effects [41]. In this study, a consistent decrease in TI value was observed in the CEO40 and CEO60 groups, indicating the potential utility of this meat in an antithrombogenic diet [17]. Previous research has indicated that USFA was more prone to react and generate unsaturated aldehydes with aromatic odors upon heating, thereby enhancing the flavor of the meat [42]. It was worth noting that duck fat was used as an ingredient in the basal diet in our study. Duck fat, rich in USFA, has been proven to have higher bioavailability in poultry in recent years [43,44]. However, increased C12:0 and ALA in the breast meat should be attributed to the supplementation of CEO, not duck fat, as duck fat only serves as one of the ingredients of the basal diet. Therefore, CEO supplementation significantly influenced the crude protein and amino acid content, as well as the fatty acid profiles, which resulted in improvements in physical characteristics and nutritional values. However, the regulatory mechanisms of thymol and carvacrol on the biosynthesis of amino acids and fatty acids in the breast muscle of broilers remain unclear and need to be further studied.

## 5. Conclusions

In this study, dietary supplementation with CEO containing thymol and carvacrol effectively enhanced meat quality by increasing muscle fiber density, augmenting antioxidant capacity, and regulating glucose metabolism in broiler meat. Furthermore, the inclusion of dietary CEO elevated crude protein and cystine content, as well as increased the proportions of C12:0 and USFA in the breast meat, thereby enhancing the nutritional value of broiler meat, which may contribute to improving human health and well-being. Notably, supplementation with 60 mg/kg of CEO exhibited the most pronounced effect on chicken meat quality in this study.

## Figures and Tables

**Table 1:** Ingredient composition and nutrient levels of basal diets (as-fed basis).

Ingredients, %	1–14 d	15–28 d	29–42 d
Corn	37.80	40.00	42.20
Soybean meal, 46% CP	25.70	23.90	22.10
Coarse rice	15.00	12.50	10.00
Wheat flour	8.00	8.00	8.00
Corn gluten meal	2.00	2.00	2.00
Cottonseed meal	4.00	4.00	4.00
Hydrolyzed feather meal	1.50	1.50	1.50
CaHPO_4_	0.90	0.85	0.80
Pulverized limestone	1.60	1.55	1.50
Duck fat	1.50	3.70	5.90
Premix ^1^	2.00	2.00	2.00
Total	100.00	100.00	100.00
Nutrients, %			
ME, MJ/kg	12.12	12.62	13.17
Crude protein	21.62	20.76	19.90
Calcium	1.14	1.10	1.06
Total phosphorus	0.53	0.51	0.49
Lysine	0.87	0.83	0.79
Methionine	0.36	0.34	0.33
Threonine	0.78	0.75	0.72

^1^ The premix provided per kg of basal diet: vitamin A, 9600 IU; vitamin D_3_, 1200 IU; vitamin E, 24 IU; vitamin K_3_, 0.60 mg; vitamin B_1_, 2.40 mg; vitamin B_2_, 9.60 mg; vitamin B_12_, 0.01 mg; pantothenic acid, 12.00 mg; niacin, 42.00 mg; biotin, 0.21 mg; folic acid, 0.66 mg; Mn (MnSO_4_·H_2_O), 120 mg; Fe (FeSO_4_·H_2_O), 100 mg; Zn (ZnSO_4_·H_2_O), 100 mg; Cu (CuSO_4_·5H_2_O), 8.00 mg; I (KIO_3_), 0.70 mg; Se (Na_2_SeO_3_), 0.30 mg.

**Table 2:** Effects of dietary supplementation with cocrystals of thymol and carvacrol on meat quality of breast muscle in broilers.

Parameter	Treatment ^1^				SEM	*p* Value
	CEO0	CEO40	CEO60	CEO80		
pH						
pH_45 min_	6.93	6.94	6.93	6.94	0.03	0.996
pH_24 h_	5.98 ^b^	6.20 ^ab^	6.45 ^a^	6.15 ^ab^	0.06	0.033
Color parameters ^2^						
L*_45 min_	45.60 ^b^	48.72 ^a^	49.20 ^a^	48.48 ^a^	0.50	0.032
a*_45 min_	2.65 ^b^	3.32 ^b^	4.35 ^a^	3.48 ^ab^	0.19	0.006
b*_45 min_	9.22	8.90	8.00	8.37	0.28	0.434
L*_24 h_	55.98	57.38	58.72	58.67	0.72	0.515
a*_24 h_	7.15	6.86	10.83	8.91	0.61	0.078
b*_24 h_	17.18	16.33	15.15	15.08	0.59	0.555
Drip loss, %	5.98	5.56	5.93	7.43	0.41	0.412
Cooking loss, %	20.23 ^a^	15.99 ^c^	17.00 ^bc^	19.55 ^ab^	0.58	0.025
Thawing loss, %	6.72	5.30	5.23	4.19	0.42	0.213
Shear force, N	53.00 ^a^	44.29 ^b^	42.69 ^b^	49.68 ^ab^	1.49	0.046

^1^ CEO0, CEO40, CEO60, and CEO80 indicate the basal diet supplemented with 0, 40, 60, and 80 mg/kg cocrystals of thymol and carvacrol, respectively. ^2^ L*, a*, and b* values represent the lightness, redness, and yellowness of the breast meat, respectively. Values are expressed as the mean and standard error of the mean (SEM). *n* = 8. ^a,b,c^ Means with different lowercase letters differ significantly among the four treatments (*p* < 0.05).

**Table 3:** Effects of dietary supplementation with cocrystals of thymol and carvacrol on chemical composition of breast muscle in broilers.

Parameter	Treatment ^1^				SEM	*p* Value
	CEO0	CEO40	CEO60	CEO80		
Moisture, %	73.67	73.67	73.50	73.63	0.22	0.993
Crude protein, %	22.65 ^b^	23.80 ^ab^	23.92 ^ab^	24.65 ^a^	0.24	0.021
Crude fat, %	0.88	0.97	0.94	0.84	0.04	0.782

^1^ CEO0, CEO40, CEO60, and CEO80 indicate the basal diet supplemented with 0, 40, 60, and 80 mg/kg cocrystals of thymol and carvacrol, respectively. Values are expressed as the mean and standard error of the mean (SEM). *n* = 8. ^a,b^ Means with different lowercase letters differ significantly among the four treatments (*p* < 0.05).

**Table 4:** Effects of dietary supplementation with cocrystals of thymol and carvacrol on the amino acid profile of breast muscle in broilers.

Parameter	Treatment ^1^				SEM	*p* Value
	CEO0	CEO40	CEO60	CEO80		
Histidine	7.78	7.42	7.45	8.04	0.14	0.327
Arginine	16.53	16.48	16.95	16.69	0.29	0.951
Serine	4.72	4.83	5.11	5.06	0.07	0.213
Glycine	10.18	10.26	10.67	10.49	0.15	0.652
Aspartic acid	17.74	17.98	18.26	18.12	0.23	0.883
Glutamic acid	35.76	36.85	38.54	37.73	0.58	0.384
Threonine	11.35	11.42	11.82	11.78	0.16	0.641
Alanine	14.31	14.38	14.69	14.79	0.17	0.718
Proline	8.76	8.77	9.22	8.99	0.13	0.595
Lysine	22.95	23.57	23.76	23.82	0.33	0.788
Cystine	4.34 ^b^	4.70 ^ab^	4.97 ^a^	4.47 ^b^	0.07	<0.01
Tyrosine	8.48	8.56	9.08	8.83	0.15	0.476
Methionine	6.38	6.30	6.57	6.24	0.13	0.842
Valine	12.66	12.33	12.33	12.61	0.15	0.809
Isoleucine	12.50	12.33	12.43	12.58	0.14	0.942
Leucine	20.44	20.27	20.81	20.86	0.25	0.810
Phenylalanine	10.12	10.21	10.35	10.47	0.13	0.800

^1^ CEO0, CEO40, CEO60, and CEO80 indicate the basal diet supplemented with 0, 40, 60, and 80 mg/kg cocrystals of thymol and carvacrol, respectively. Values are expressed as the mean and standard error of the mean (SEM). *n* = 8. ^a,b^ Means with different lowercase letters differ significantly among the four treatments (*p* < 0.05).

**Table 5:** Effects of dietary supplementation with cocrystals of thymol and carvacrol on the fatty acid profile of breast muscle in broilers.

Parameter	Treatment ^1^				SEM	*p* Value
	CEO0	CEO40	CEO60	CEO80		
Fatty acid composition (% total fatty acid)						
C8:0	0.03	0.02	0.02	0.03	0.002	0.271
C12:0	0.012 ^c^	0.017 ^a^	0.016 ^ab^	0.014 ^bc^	0.001	0.009
C14:0	0.31	0.38	0.38	0.36	0.013	0.173
C15:0	0.036	0.052	0.049	0.045	0.002	0.080
C16:0	24.04	25.11	24.53	23.70	0.293	0.374
C17:0	0.07	0.07	0.07	0.08	0.002	0.665
C18:0	14.89	11.31	11.97	13.12	0.604	0.165
C20:0	0.10	0.08	0.08	0.10	0.004	0.168
C21:0	0.04	0.02	0.03	0.03	0.003	0.442
C22:0	0.05	0.04	0.04	0.05	0.003	0.245
C23:0	0.04	0.03	0.03	0.03	0.003	0.425
C24:0	0.02	0.02	0.02	0.02	0.002	0.441
Total SFA ^2^	39.64	37.15	37.24	37.58	0.396	0.074
C16:1	2.19	3.66	3.04	2.65	0.262	0.243
C18:1n9c	30.76	37.34	35.70	34.33	1.252	0.302
C20:1	0.54	0.52	0.56	0.59	0.026	0.831
Total MUFA ^3^	33.48	41.52	39.30	37.57	1.472	0.271
C18:2n6c	16.75	14.85	16.23	16.29	0.411	0.412
C18:3n6 (GLA)	0.09	0.09	0.10	0.09	0.004	0.687
C18:3n3 (ALA)	0.32 ^b^	0.38 ^a^	0.38 ^a^	0.38 ^a^	0.009	0.030
C20:2	1.22	0.87	0.95	1.14	0.088	0.488
C20:3n6	1.75	1.32	1.39	1.61	0.119	0.593
C20:4n6	6.09	3.42	3.94	4.79	0.514	0.288
C20:5n3 (EPA)	0.22	0.15	0.16	0.21	0.018	0.426
C22:6 (DHA)	0.45	0.26	0.32	0.34	0.038	0.392
n − 3 PUFA ^4^	0.99	0.79	0.86	0.93	0.048	0.539
n − 6 PUFA ^5^	24.26	20.08	22.25	23.67	0.828	0.295
Total PUFA ^6^	26.88	21.34	23.47	24.85	1.131	0.383
Total USFA ^7^	59.65 ^b^	61.92 ^a^	62.86 ^a^	61.56 ^b^	0.384	0.001
USFA:SFA	1.53	1.70	1.69	1.66	0.027	0.084
h/H ^8^	2.42	2.33	2.40	2.34	0.031	0.675
AI ^9^	0.41	0.42	0.41	0.42	0.005	0.762
TI ^10^	1.23 ^a^	1.10 ^b^	1.10 ^b^	1.19 ^a^	0.016	0.002

^1^ CEO0, CEO40, CEO60, and CEO80 indicate the basal diet supplemented with 0, 40, 60, and 80 mg/kg cocrystals of thymol and carvacrol, respectively. ^2^ Total SFA (saturated fatty acids) = C8:0 + C12:0 + C14:0 + C16:0 + C17:0 + 18:0 + C20:0 + C21:0 + C22:0 + C23:0 + C24:0. ^3^ Total MUFA (unsaturated fatty acids) = C16:1 + C18:1n9c + C20:1. ^4^ n − 3 PUFA (polyunsaturated fatty acids) = C18:3n3 + C20:5n3 + C22:6. ^5^ n − 6 PUFA = C18:2n6c + C18:3n6 + C20:3n6 + C20:4n6. ^6^ Total PUFA = C18:2n6c + C18:3n6 + C18:3n3 + C20:2 + C20:3n6 + C20:4n6 + C20:5n3 + C22:6. ^7^ Total USFA = total MUFA + total PUFA. ^8^ h/H (ratio between hypocholesterolemic and hypercholesterolemic fatty acids) = (C18:1n9c + total PUFA)/(C14:0 + C16:0). ^9^ AI (atherogenocity index) = (C12:0 + 4 × C14:0 + C16:0)/(total MUFA + total PUFA). ^10^ TI (thrombogenicity index) = (C14:0 + C16:0 + C18:0)/(0.5 × total MUFA + 3 × n − 3 PUFA + 0.5 × n − 6 PUFA + n − 3 PUFA/n − 6 PUFA). Values are expressed as the mean and standard error of the mean (SEM). *n* = 8. ^a,b,c^ Means with different lowercase letters differ significantly among the four treatments (*p* < 0.05).

## Data Availability

The original contributions presented in the study are included in the article, further inquiries can be directed to the corresponding author.

## References

[1] Kiyma Z., Küçükyilmaz K., Çetinkaya M., Ateş A., Atalay H., Akdağ A., Bozkurt M., Gürsel F.E. (2017). Effect of Lavender (*Lavandula stoechas*) Essential Oil on Growth Performance, Carcass Characteristics, Meat Quality and Antioxidant Status of Broilers. S. Afr. J. Anim. Sci..

[2] Hartcher K.M., Lum H.K. (2020). Genetic Selection of Broilers and Welfare Consequences: A Review. World’s Poult. Sci. J..

[3] Puvača N., Tufarelli V., Giannenas I. (2022). Essential Oils in Broiler Chicken Production, Immunity and Meat Quality: Review of Thymus Vulgaris, Origanum Vulgare, and Rosmarinus Officinalis. Agriculture.

[4] Hernández-Coronado A.C., Silva-Vázquez R., Rangel-Nava Z.E., Hernández-Martínez C.A., Kawas-Garza J.R., Hume M.E., Méndez-Zamora G. (2019). Mexican Oregano Essential Oils given in Drinking Water on Performance, Carcass Traits, and Meat Quality of Broilers. Poult. Sci..

[5] Mazzeo P.P., Carraro C., Monica A., Capucci D., Pelagatti P., Bianchi F., Agazzi S., Careri M., Raio A., Carta M. (2019). Designing a Palette of Cocrystals Based on Essential Oil Constituents for Agricultural Applications. ACS Sustain. Chem. Eng..

[6] Llana-Ruiz-Cabello M., Pichardo S., Maisanaba S., Puerto M., Prieto A.I., Gutiérrez-Praena D., Jos A., Cameán A.M. (2015). In Vitro Toxicological Evaluation of Essential Oils and Their Main Compounds Used in Active Food Packaging: A Review. Food Chem. Toxicol..

[7] Luna A., Lábaque M.C., Zygadlo J.A., Marin R.H. (2010). Effects of Thymol and Carvacrol Feed Supplementation on Lipid Oxidation in Broiler Meat. Poult. Sci.

[8] Stevanović Z.D., Bošnjak-Neumüller J., Pajić-Lijaković I., Raj J., Vasiljević M. (2018). Essential Oils as Feed Additives—Future Perspectives. Molecules.

[9] Qiao N., Li M., Schlindwein W., Malek N., Davies A., Trappitt G. (2011). Pharmaceutical Cocrystals: An Overview. Int. J. Pharm..

[10] Clark D.L., Velleman S.G. (2016). Spatial Influence on Breast Muscle Morphological Structure, Myofiber Size, and Gene Expression Associated with the Wooden Breast Myopathy in Broilers. Poult. Sci..

[11] Gao H., Zhang Y., Liu K., Fan R., Li Q., Zhou Z. (2022). Dietary Sodium Butyrate and/or Vitamin D3 Supplementation Alters Growth Performance, Meat Quality, Chemical Composition, and Oxidative Stability in Broilers. Food Chem..

[12] Yan E., Wang Y., He L., Guo J., Zhang X., Yin J. (2022). Effects of Dietary L-Malic Acid Supplementation on Meat Quality, Antioxidant Capacity and Muscle Fiber Characteristics of Finishing Pigs. Foods.

[13] Yang X., Zhang B., Guo Y., Jiao P., Long F. (2010). Effects of Dietary Lipids and Clostridium Butyricum on Fat Deposition and Meat Quality of Broiler Chickens. Poult. Sci..

[14] Zhuang H., Savage E.M., Smith D.P., Berrang M.E. (2008). Effect of Dry-Air Chilling on Warner-Bratzler Shear Force and Water-Holding Capacity of Broiler Breast Meat Deboned Four Hours Postmortem. Int. J. Poult. Sci..

[15] Jin S., Pang Q., Yang H., Diao X., Shan A., Feng X. (2021). Effects of Dietary Resveratrol Supplementation on the Chemical Composition, Oxidative Stability and Meat Quality of Ducks (*Anas platyrhynchos*). Food Chem..

[16] Chen J., Li F., Yang W., Jiang S., Li Y. (2021). Supplementation with Exogenous Catalase from Penicillium Notatum in the Diet Ameliorates Lipopolysaccharide-Induced Intestinal Oxidative Damage through Affecting Intestinal Antioxidant Capacity and Microbiota in Weaned Pigs. Microbiol. Spectr..

[17] Anacleto P., Maulvault A.L., Bandarra N.M., Repolho T., Nunes M.L., Rosa R., Marques A. (2014). Effect of Warming on Protein, Glycogen and Fatty Acid Content of Native and Invasive Clams. Food Res. Int..

[18] Mancini R.A., Hunt M.C. (2005). Current Research in Meat Color. Meat Sci..

[19] Gholami-Ahangaran M., Ahmadi-Dastgerdi A., Azizi S., Basiratpour A., Zokaei M., Derakhshan M. (2021). Thymol and Carvacrol Supplementation in Poultry Health and Performance. Vet. Med. Sci..

[20] Hashemipour H., Kermanshahi H., Golian A., Veldkamp T. (2013). Effect of Thymol and Carvacrol Feed Supplementation on Performance, Antioxidant Enzyme Activities, Fatty Acid Composition, Digestive Enzyme Activities, and Immune Response in Broiler Chickens. Poult. Sci..

[21] Garcia-Galicia I.A., Arras-Acosta J.A., Huerta-Jimenez M., Rentería-Monterrubio A.L., Loya-Olguin J.L., Carrillo-Lopez L.M., Tirado-Gallegos J.M., Alarcon-Rojo A.D. (2020). Natural Oregano Essential Oil May Replace Antibiotics in Lamb Diets: Effects on Meat Quality. Antibiotics.

[22] Wang B., Li H., Huang Z., Kong B., Liu Q., Wang H., Xu M., Xia X. (2021). Dynamic Changes in the Qualities and Heterocyclic Aromatic Amines of Roasted Pork Induced by Frying Temperature and Time. Meat Sci..

[23] Galli G.M., Gerbet R.R., Griss L.G., Fortuoso B.F., Petrolli T.G., Boiago M.M., Souza C.F., Baldissera M.D., Mesadri J., Wagner R. (2020). Combination of Herbal Components (Curcumin, Carvacrol, Thymol, Cinnamaldehyde) in Broiler Chicken Feed: Impacts on Response Parameters, Performance, Fatty Acid Profiles, Meat Quality and Control of Coccidia and Bacteria. Microb. Pathog..

[24] Ramos F.A., Martínez A.P., Montes E.S., Gaytán C.N., Cázarez A.S.H., Tovar J.C., Sánchez J.G., del Carmen Rodríguez Castillo J. (2017). El uso de aceite de orégano en la dieta del pollo de engorde incrementa la acumulación de timol y carvacrol en carne de pechuga. Acta Univ..

[25] Jahani M., Pira M., Aminifard M.H. (2020). Antifungal Effects of Essential Oils against *Aspergillus niger* in Vitro and in Vivo on Pomegranate (*Punica granatum*) Fruits. Sci. Hortic..

[26] Chhimpa N., Singh N., Puri N., Kayath H.P. (2023). The Novel Role of Mitochondrial Citrate Synthase and Citrate in the Pathophysiology of Alzheimer’s Disease. J. Alzh. Dis..

[27] Leek B.T., Mudaliar S.R.D., Henry R., Mathieu-Costello O., Richardson R.S. (2001). Effect of Acute Exercise on Citrate Synthase Activity in Untrained and Trained Human Skeletal Muscle. Am. J. Physiol. Regul. Integr. Comp. Physiol..

[28] Scheffler T.L., Matarneh S.K., England E.M., Gerrard D.E. (2015). Mitochondria Influence Postmortem Metabolism and pH in an in Vitro Model. Meat Sci..

[29] Zhang C., Yang L., Zhao X., Chen X., Wang L., Geng Z. (2018). Effect of Dietary Resveratrol Supplementation on Meat Quality, Muscle Antioxidative Capacity and Mitochondrial Biogenesis of Broilers. J. Sci. Food Agric..

[30] Škorpilová T., Šístková I., Adamcová M., Pohůnek V., Kružík V., Ševčík R. (2019). Measuring Citrate Synthase Activity as an Enzymatic Approach to the Differentiation of Chilled and Frozen/Thawed Meat. Meat Sci..

[31] Rimini S., Petracci M., Smith D.P. (2014). The Use of Thyme and Orange Essential Oils Blend to Improve Quality Traits of Marinated Chicken Meat. Poult. Sci..

[32] Alfaig, Ebrahim, Angelovicova M., Kral M., Vietoris V., Zidek R. (2013). Effect of Probiotics and Thyme Essential Oil on the Texture of Cooked Chicken Breast Meat. Acta Sci. Pol. Technol. Aliment..

[33] Zhao G.P., Cui H.X., Liu R.R., Zheng M.Q., Chen J.L., Wen J. (2011). Comparison of Breast Muscle Meat Quality in 2 Broiler Breeds. Poult. Sci..

[34] Starčević K., Krstulović L., Brozić D., Maurić M., Stojević Z., Mikulec Ž., Bajić M., Mašek T. (2015). Production Performance, Meat Composition and Oxidative Susceptibility in Broiler Chicken Fed with Different Phenolic Compounds. J. Sci. Food Agric..

[35] İpçak H.H., Alçiçek A. (2018). Addition of Capsicum Oleoresin, Carvacrol, Cinnamaldehyde and Their Mixtures to the Broiler Diet II: Effects on Meat Quality. J. Anim. Sci. Technol..

[36] Zhu X., Liu W., Yuan S., Chen H. (2014). The Effect of Different Dietary Levels of Thyme Essential Oil on Serum Biochemical Indices in Mahua Broiler Chickens. Ital. J. Anim. Sci..

[37] Zhou R., Grant J., Goldberg E.M., Ryland D., Aliani M. (2019). Investigation of Low Molecular Weight Peptides (<1 kDa) in Chicken Meat and Their Contribution to Meat Flavor Formation. J. Sci. Food Agr..

[38] Mizugaki A., Kato H., Takeda T., Inoue Y., Hasumura M., Hasegawa T., Murakami H. (2022). Cystine Reduces Mitochondrial Dysfunction in C2C12 Myotubes under Moderate Oxidative Stress Induced by H_2_O_2_. Amino Acids.

[39] Khan H.U., Aamir K., Sisinthy S.P., Nagojappa N.B.S., Arya A. (2020). Food Additive “Lauric Acid” Possess Non-Toxic Profile on Biochemical, Haematological and Histopathological Studies in Female Sprague Dawley (SD) Rats. PeerJ.

[40] Hoa V.B., Song D.H., Seol K.H., Kang S.M., Kim H.W., Kim J.H., Cho S.H. (2022). Coating with Chitosan Containing Lauric Acid (C12:0) Significantly Extends the Shelf-Life of Aerobically—Packaged Beef Steaks during Refrigerated Storage. Meat Sci..

[41] Kim K.B., Nam Y.A., Kim H.S., Hayes A.W., Lee B.M. (2014). α-Linolenic Acid: Nutraceutical, Pharmacological and Toxicological Evaluation. Food Chem. Toxicol..

[42] Sun X., Zhang B., Han J., Wei C., Liu W. (2022). Effect of Roasting Temperature and Time on Volatile Compounds, Total Tocopherols, and Fatty Acids of Flaxseed Oil. J. Food Sci..

[43] Ao X., Kim I.H. (2020). Effects of dietary lipid sources on growth performance and carcass traits in Pekin ducks. Poult. Sci..

[44] Zeng X., Zhang K., Tian G., Ding X., Bai S., Wang J., Lv L., Liao Y., Xuan Y., Zeng Q. (2022). Effects of fat pre-emulsification on the growth performance, serum biochemical index, digestive enzyme activities, nutrient utilization, and standardized ileal digestibility of amino acids in Pekin ducks fed diets with different fat sources. Animals.

